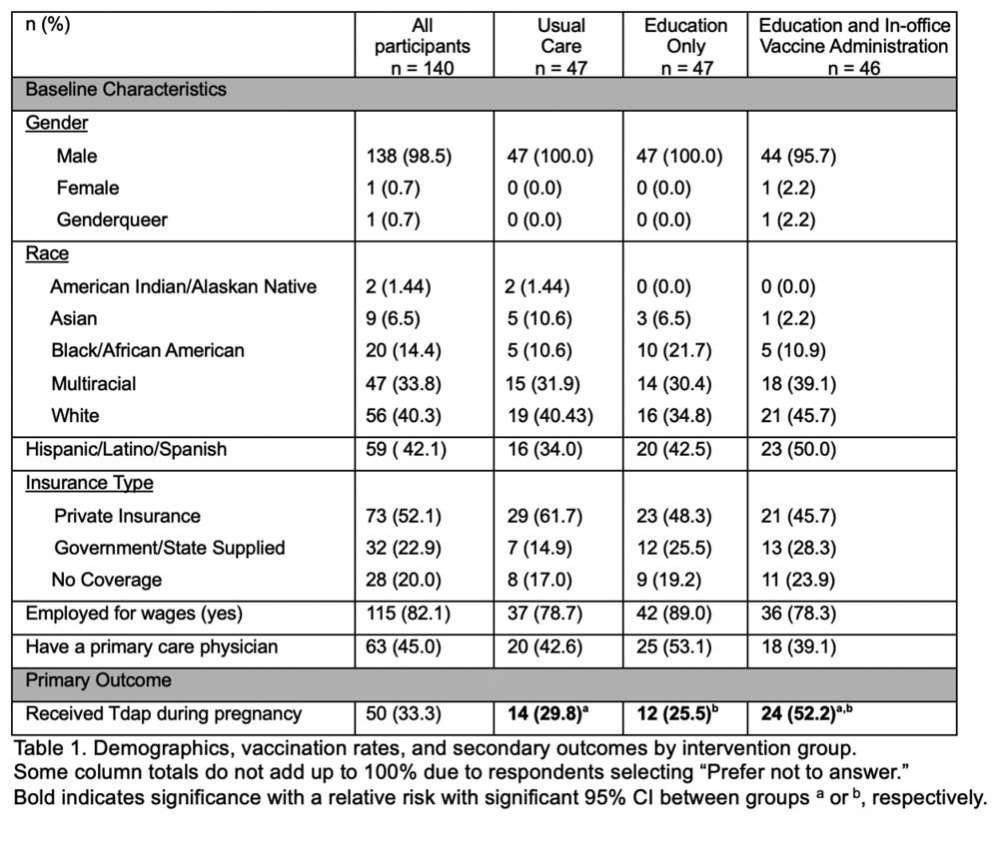# Oral Plenary Session 2 – Fellows Plenary: Thursday, February 12, 2026 8:00 AM – 10:00 AM

**DOI:** 10.1002/pmf2.70163

**Published:** 2026-02-09

**8:00 AM – 8:15 AM**

## 37 | Antepartum Magnesium Sulfate Before Delivery at 22–24 Weeks Gestation and Long‐Term Neurodevelopmental Outcomes

Margaret R. Page^1^; Barbara Do^2^; Namasivayam Ambalavanan^3^; Colm P. Travers^4^; Waldemar A. Carlo^5^; Samuel Gentle^3^; Amelia Schuyler^3^; Akila Subramaniam^1^; Ashley N. Battarbee^1^

On behalf of the Eunice Kennedy Shriver NICHD Neonatal Research Network

^1^Center for Research in Women's Health, University of Alabama at Birmingham, Birmingham, AL; ^2^RTI International, Research Triangle Park, NC; ^3^University of Alabama at Birmingham Heersink School of Medicine, Birmingham, AL; ^4^Department of Pediatrics, University of Alabama at Birmingham Heersink School of Medicine, Birmingham, AL; ^5^Department of Pediatrics, University of Alabama at Birmingham, Birmingham, AL

**Objective**: SMFM and ACOG recommend magnesium sulfate for neuroprotection before delivery at 24 0/7–31 6/7 weeks and advise consideration at 23 weeks. At 22 weeks, magnesium sulfate is not recommended although corticosteroids can be considered. We aimed to evaluate the association between in utero magnesium exposure and 2‐year neurodevelopmental outcomes after periviable delivery.

**Study Design**: Multicenter cohort study of prospectively collected data of children born at 22 0/7–24 6/7 weeks from 2012–2022 in NICHD Neonatal Research Network hospitals and had 2‐year follow‐up. Children with congenital anomalies, death at <12 postnatal hours, and from high‐order multiple gestation were excluded. The primary outcome was severe neurodevelopmental impairment (NDI) or death at 2 years (Table 1). Baseline characteristics and outcomes were compared between those exposed to magnesium versus not exposed. Multilevel logistic regression estimated the association between magnesium exposure and outcomes with tests for effect modification by delivery gestational age (GA).

**Results**: Of 4,179 included children, 3242 (78%) were exposed to magnesium and 937 (22%) were not exposed. Children exposed to magnesium were more likely to have maternal private insurance, antenatal corticosteroid and antibiotic exposure, cesarean delivery, and later delivery GA. Children exposed to magnesium had lower rates of severe NDI or death (63% vs 70%, *p* < 0.01) and other outcomes (Table 1). These associations were not significant after adjusting for covariates (aOR 1.00, CI 0.81–1.25), but significance could be restored with exclusion of corticosteroids (aOR 0.78, CI 0.65–0.95; Table 1). The lack of association between magnesium and NDI or death was consistent across all delivery GA (Table 2).

**Conclusion**: In utero magnesium exposure before delivery at 22 0/7–24 6/7 weeks was not independently associated with better neurodevelopmental outcomes at 2 years after accounting for antenatal corticosteroid receipt. Management of pregnant patients at risk of periviable delivery should prioritize antenatal corticosteroid administration.

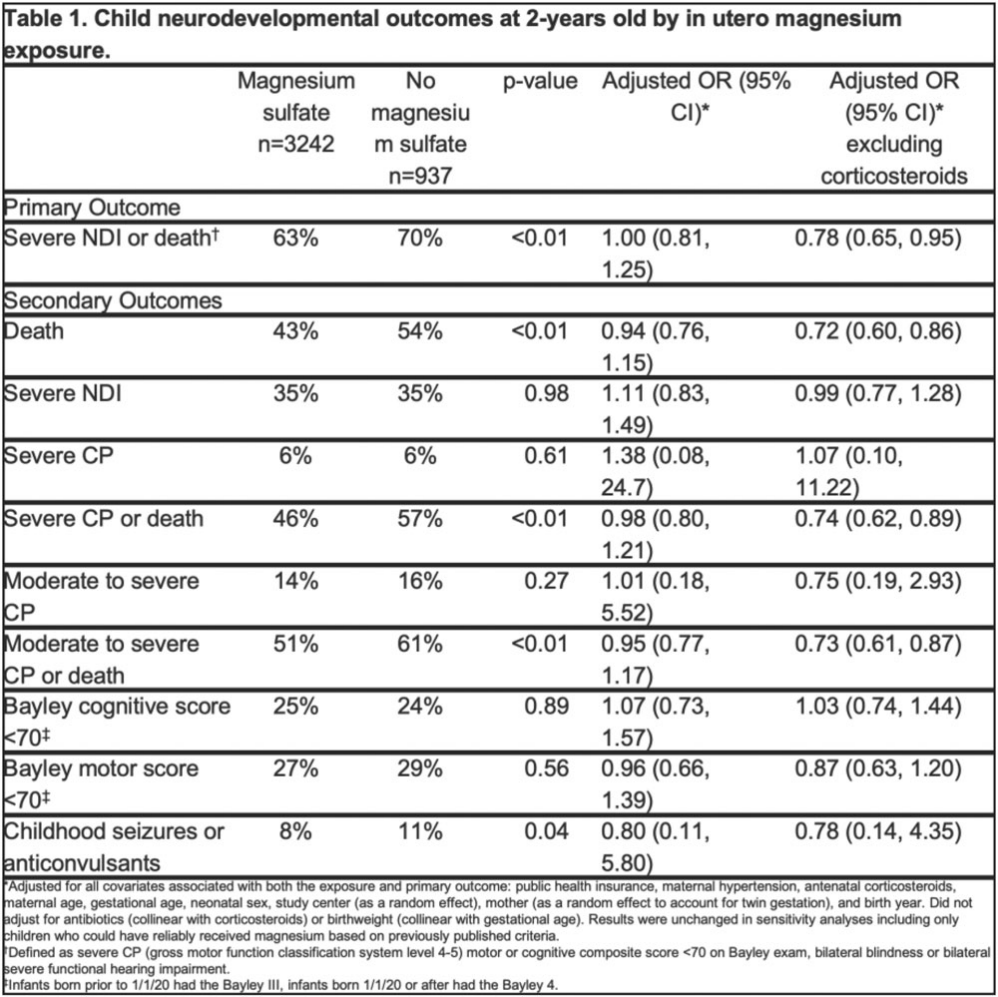

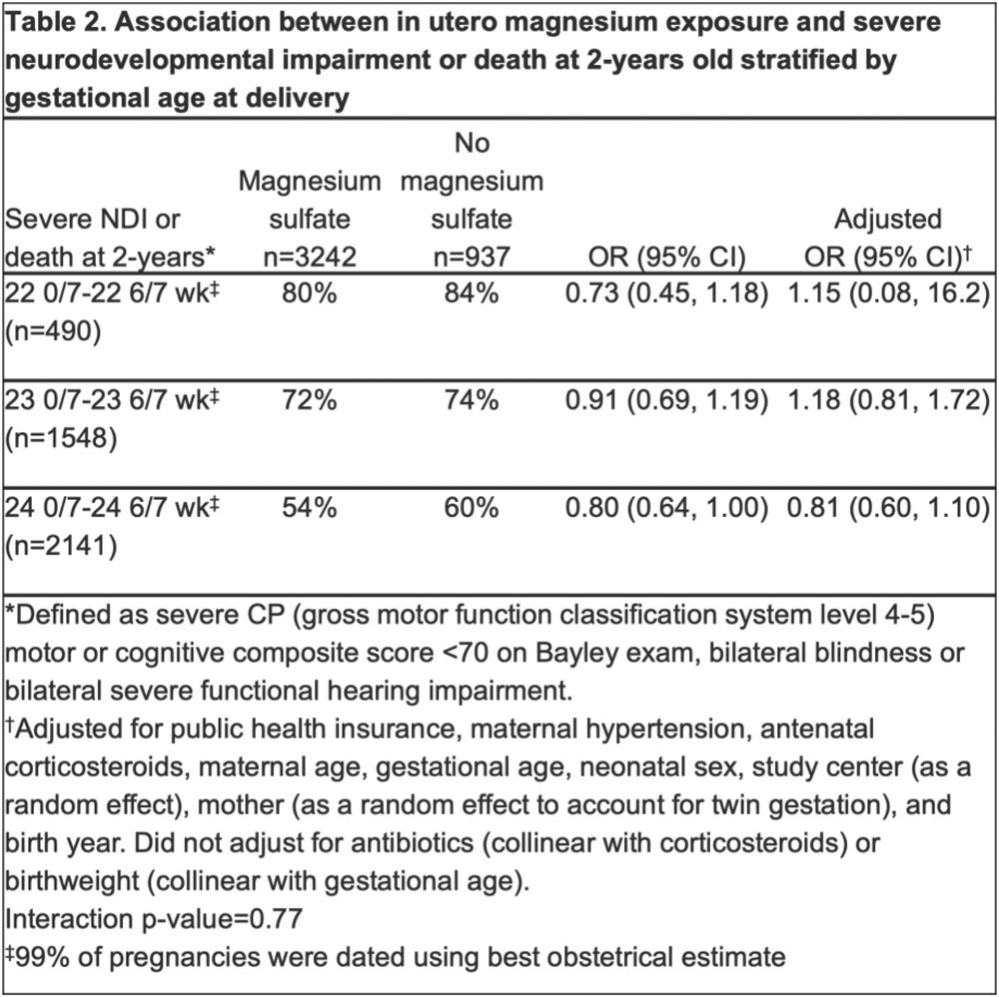

**8:15 AM – 8:30 AM**

## 38 | **Repeat Type and Screen Testing in Antepartum Admissions: Where Is the Evidence?**

Kimen S. Balhotra^1^; Aaron W. Roberts^2^; Farah H. Amro^1^; Baha M. Sibai^1^

^1^Division of Maternal‐Fetal Medicine, Department of Obstetrics, Gynecology and Reproductive Sciences, McGovern Medical School at UTHealth Houston, Houston, TX; ^2^Department of Obstetrics, Gynecology and Reproductive Sciences, McGovern Medical School at UTHealth Houston, Houston, TX

**Objective**: The 72‐hour repeat type and screen (T&S) practice in inpatient hospitalized obstetrics patients is based on guidelines from Association for the Advancement of Blood & Biotherapies with no data to support its use in pregnant patients. In hospitalized patients without bleeding, new clinically significant alloantibodies are exceedingly rare. This study evaluated the incidence of newly detected alloantibodies after an initial negative screen and calculated the financial cost of routine repeat testing.

**Study Design**: This was a retrospective review of obstetric hospitalizations from July 2020 to October 2024, who were ≥24 weeks gestation, had a hospital stay greater than ≥72 hours, and had ≥2 T&S tests performed. All obstetric hospitalizations regardless of indication were included such as, hypertensive disorders of pregnancy, preterm labor, preterm premature rupture of membranes, fetal indication for admission, placenta previa, and placenta accreta spectrum. CPT codes were collected for T&S, crossmatch, blood transfusion, and antibody identification. First and last T&S were compared. The itemized cost‐estimation for testing was derived from the hospital charge master which by law is publicly available.

**Results**: A total of 107,718 patients accounting for 128,476 obstetric hospitalizations were included. The length of stay among of patients is summarized in Table 1. Among them, 2472 (2.2%) had a positive antibody screen, of which all were detected on admission. Of the positives, 1.13% (*n* = 28) were anti‐D, 0.24% (*n* = 6) were non‐Rh D, and 98.6% (*n* = 2,438) were due to Rh‐D immune globulin. After admission, 62,587 additional T&S tests were performed with no new antibody detections (0/62,587). The estimated cost of excess testing was $60,099,166 with a monthly cost of $1,178,415 and a daily cost of $39,280 (Figure 1).

**Conclusion**: This study highlights the high cost and low yield of routine repeat T&S testing in obstetric patients. This large cohort of >100,000 pregnant patients, supports re‐evaluating the practice of routine repeat T&S tests in hospitalized patients with obstetric complications.

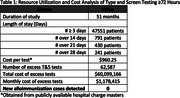

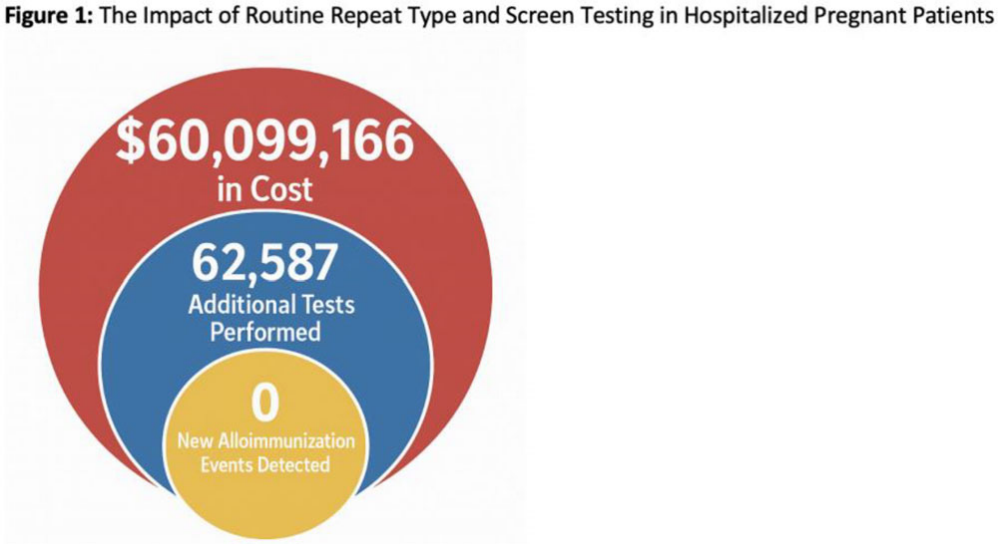

**8:30 AM – 8:45 AM**

## 39 | **AI Based Ultrasound Screening for Early, Accurate Identification of Placenta Accreta Spectrum**

Alexandra L. Hammerquist^1^; Sanmay Sarada^2^; Yamely H. Mendez^1^; Christina C. Reed^1^; Enrico R. Barrozo^3^; Jessian L. Munoz^4^; Wesley Lee^2^; Amir A. Shamshirsaz^5^; Michael A. Belfort^1^; Michael D. Jochum, Jr.^1^; Hendrik A. Lombaard^1^

^1^Baylor College of Medicine, Houston, TX; ^2^Baylor College of Medicine, Baylor College of Medicine, TX; ^3^Baylor College of Medicine and Texas Children's Hospital, Houston, TX; ^4^Texas Children's Hospital and Baylor College of Medicine, Houston, TX; ^5^Division of Maternal‐Fetal Medicine, Department of Obstetrics and Gynecology, Baylor College of Medicine, Houston, TX

**Objective**: Placenta Accreta Spectrum (PAS) is a leading cause of maternal morbidity and mortality and is increasing in incidence, yet only 30% of cases are diagnosed antenatally. While ultrasonographic findings may inform risk of PAS, multiple factors may lead to an inconclusive or misdiagnosis. We hypothesized that a novel artificial intelligence (AI) model could be used as a screening tool to accurately predict PAS by interpreting 2D ultrasonographic placental images.

**Study Design**: This single‐center retrospective study included 756 placental ultrasound DICOM files from which 38,907 grayscale PNG frames were extracted from 113 patients at risk for PAS from 2018 to 2025. Mean gestational age at ultrasound was 30.89 ± 3.67 weeks. Patients were stratified to produce 79/17/17 train/validation/test groups. Images were classified by final pathologic FIGO grades. We used an ImageNet pretrained EfficientNetB0 backbone with global average pooling and regularized sigmoid head. Frame level probabilities from the convolutional neural network (CNN) output were averaged to patient level consensus scores (Figure 1A). These scores, together with number of prior Cesarean sections and previa status were input as variables into training a logistic regression, random forest, and gradient boosting classifier which were ensembled for final prediction of PAS incorporating patient identifiable risk factors (Figure 1B).

**Results**: The CNN ensembled model predicted the presence or absence of PAS accurately in 88% of cases with a sensitivity of 100% and specificity of 75%. The positive predictive value was 81.8% and the negative predictive value was 100%. There were no false negatives in the testing cohort (Figure 1C). The AUC‐ROC was 0.972 (Figure 1D). Model variable importance scores concentrated at the placental interface, highlighting biological plausibility (Figure 2).

**Conclusion**: This novel AI model robustly achieved accurate, sensitive PAS prediction before delivery. The results of the model support its use as a screening tool to reduce missed diagnoses in PAS screening, warranting future prospective trials.

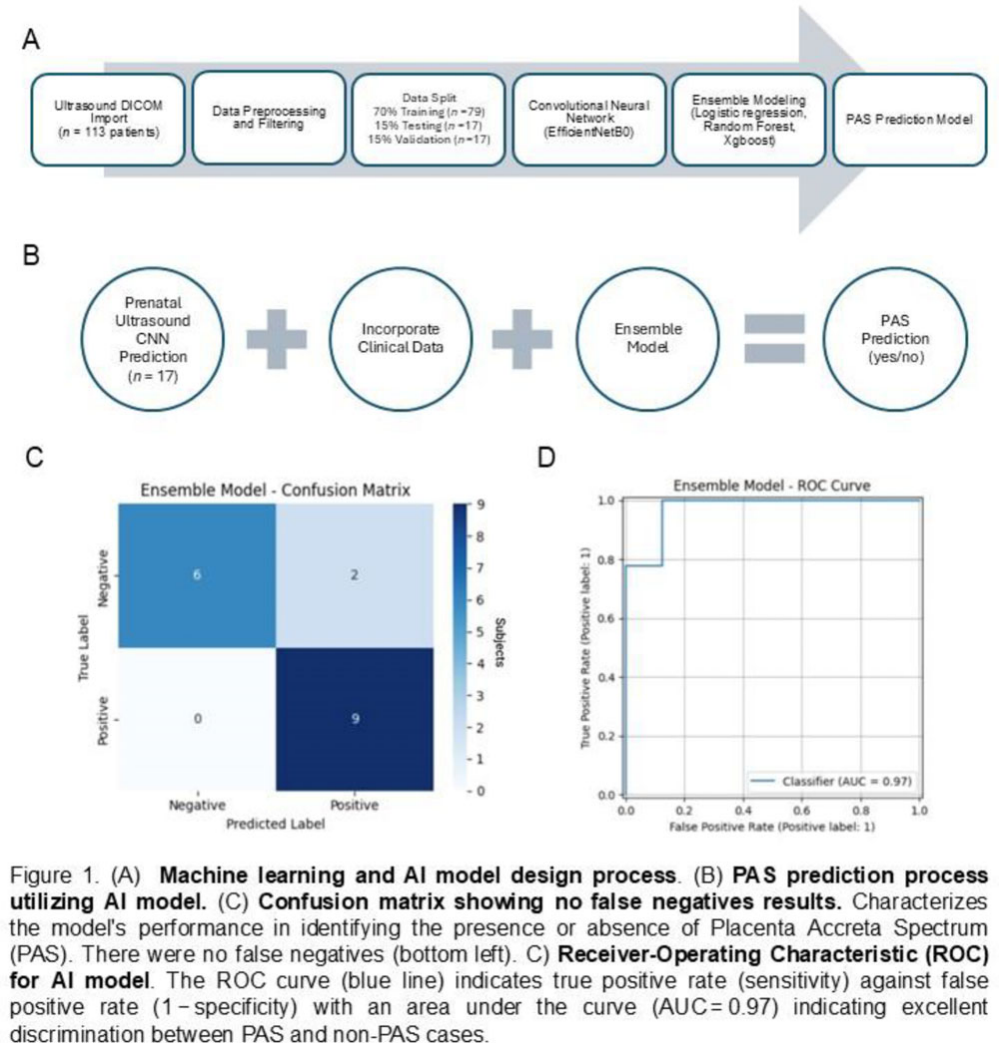

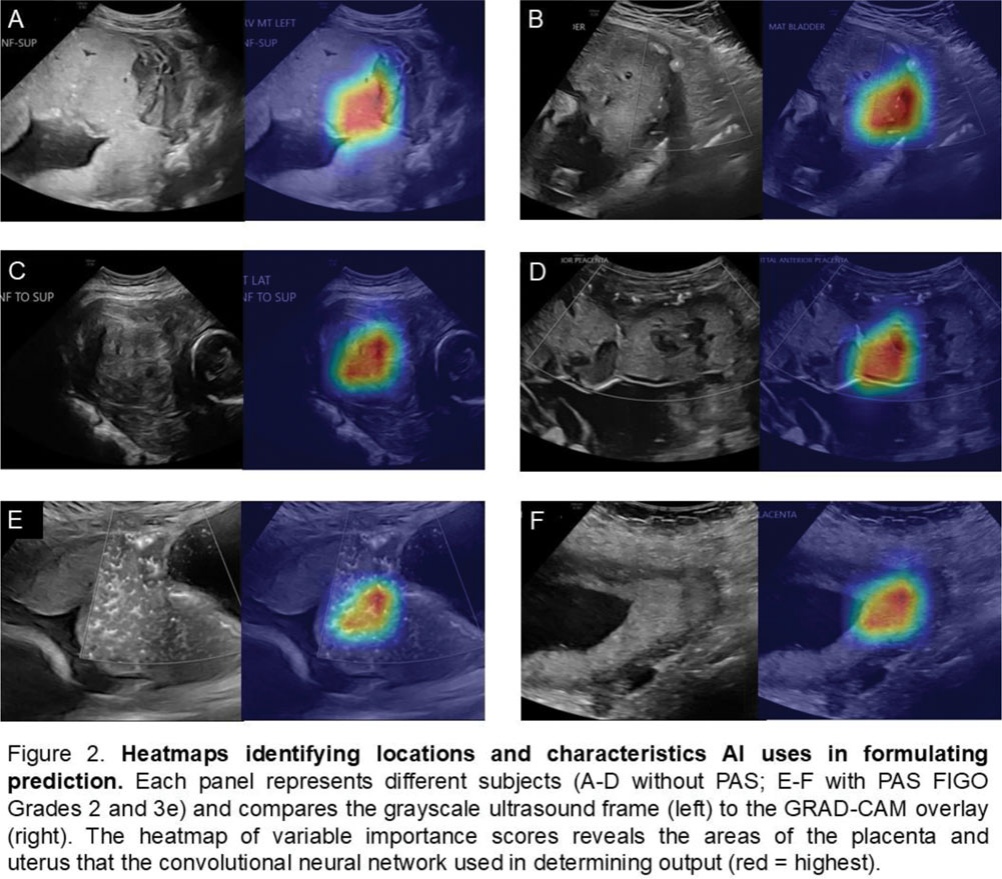

**8:45 AM – 9:00 AM**

## 40 | **Intraplacental Gene Transfer of IGF‐1 Corrects Preeclampsia in BPH/5 Mouse**

Kristen L. Moriarty^1^; Sanjukta Majumder^2^; Jennifer Sones^3^; Timothy Crombleholme^4^

^1^UConn Health, Farmington, CT; ^2^Connecticut Children's Medical Center, Farmington, CT; ^3^University of Colorado, Fort Collins, CO; ^4^Connecticut Children's Medical Center, Hartford, CT

**Objective**: Preeclampsia (PE) causes serious maternal and fetal complications, with mechanisms still unclear. The BPH/5 is the only spontaneous mouse model that mimics human features of PE. We hypothesize that placental IGF‐1 gene transfer could correct endothelial dysfunction and the maternal PE in pregnant BPH/5.

**Study Design**: Treatment groups (N): C57 Ad‐LacZ (8), C57 Ad‐IGF‐1 (8), BPH/5 Ad‐LacZ (4), and BPH/5 Ad‐IGF‐1 (8). Timed‐mated dams were acclimated to tail‐cuff BP. At laparotomy embryonic day 16, (e16), all placentas received 1 × 10⁸ PFU of adenovirus with human IGF‐1 (Ad‐IGF‐1) OR a control (Ad‐LacZ). Delivery at E20‐21 allowed for placentas, maternal serum, liver, kidney, and litter outcomes. Total urine protein was assessed across gestational timepoints. Placental microvascular density (MVD) was quantified (CD31, ImageJ). Maternal liver and kidney were stained with H&E and PAS for steatosis and glomerulosclerosis. Serum sFlt‐1 was measured by ELISA.

**Results**: In BPH/5 mice, SBP (*p* = 0.0001), MAP (*p* = 0.001), and DBP (*p* = 0.03) were significantly lower following Ad‐IGF‐1 vs. Ad‐LacZ. Urine protein differed across BPH/5 groups (*p* = <0.05), with a trend toward reduction post‐treatment (*p* = 0.0635) and significant decrease vs. non‐pregnant mice (*p* = 0.0492). There was no difference between groups in pup weight, demise rate, or reabsorption. Glomerulosclerosis was reduced in BPH/5 Ad‐IGF‐1 (*p* = 0.0255; 62.97% decrease, η^2^ = 0.48). sFlt‐1 levels were unchanged between groups (*p* = 0.70). Placental MVD (*p* = 0.0031), vessel count (VC) (*p* = 0.0005), and particle size (PS) (*p* = 0.0026) differed significantly in groups. Ad‐IGF‐1 increased MVD (*p* = 0.0122), reduced VC (*p* = 0.0014), and increased PS (*p* = 0.0014) in BPH/5.

**Conclusion**: Intraplacental gene transfer of IGF‐1 in BPH/5 restored MVD, normalized VC, increased PS, improved BP, proteinuria, and glomerulosclerosis without affecting fetal viability. Correction of PE in BPH/5 by IGF‐1 appears to be mediated by expansion of the placental microvasculature. Intraplacental IGF‐1 gene transfer corrects PE in the BPH/5 mice and may offer insight into an effective treatment for severe PE.

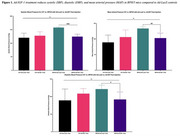

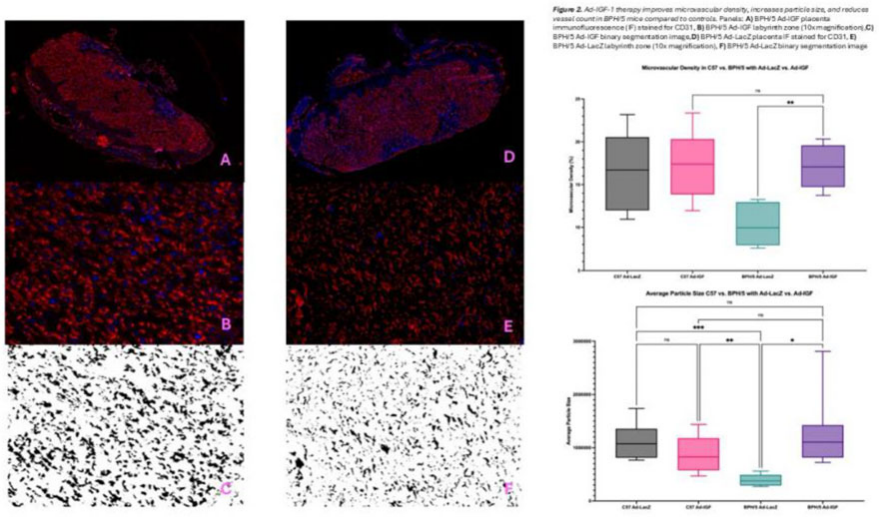

**9:00 AM – 9:15 AM**

## 41 | **Dermabond Prineo Versus Subcuticular Suture for Cesarean Delivery Skin Closure: A Randomized‐Controlled Trial**

Kristine Brown^1^; Mariam Ayyash^1^; Hooman A. Azad^2^; Lielle Yellin^1^; Olivia F. Schulist^1^; Shai Bejerano^1^; Helen B. Gomez Slagle^1^; Said Saab^1^; Jean‐Ju Sheen^3^; Russell S. Miller^4^; Uma M. Reddy^1^; Lilly Liu^5^

^1^Columbia University Irving Medical Center, New York, NY; ^2^Columbia University, Columbia University, NY; ^3^New York Presbyterian, Columbia University Irving Medical Center, Northvale, NJ; ^4^Division of Maternal‐Fetal Medicine, Department of Obstetrics and Gynecology, Columbia University Irving Medical Center, New York, NY; ^5^Albert Einstein School of Medicine/Montefiore Medical Center, New York, NY

**Objective**: While surgical adhesive dressings have been evaluated in non‐obstetric surgery, the optimal incision closure method for cesarean delivery (CD) remains unknown. We sought to evaluate patient scar satisfaction following surgical adhesive skin closure in non‐urgent cesarean delivery.

**Study Design**: This was a randomized‐controlled trial of Dermabond Prineo adhesive system vs. standard delayed‐absorbable subcuticular suture closure for CD. Patients with an adhesive allergy, cesarean hysterectomy, emergent delivery, or a vertical skin incision were excluded. The primary outcome was patient satisfaction with their scar, assessed 6 weeks postoperatively by the validated Patient Scar Assessment Scale (PSAS). Secondary outcomes included operative time, pain scores, wound complications, and hospital readmission rates.  A sample size of 188 subjects was calculated to achieve 80% power while assuming up to 20% loss to follow up. Randomization was 1:1 and stratified by pre‐delivery BMI (BMI <30 vs. ≥30). Dermabond Prineo was applied following manufacturer instructions. Surgical techniques and choice of suture were at the discretion of the obstetrical providers. Analysis was by intention to treat.

**Results**: 188 patients were enrolled and 37 lost to follow up, resulting in 151 patients included for analysis. Baseline and delivery characteristics were similar between groups (Table 1) except for higher mean maternal age in the Dermabond group. 4‐0 monofilament suture was used for closure in 97.1% of subjects within the suture group. The majority of patients in both groups underwent repeat pre‐labor CD. There was no difference in patient scar satisfaction between groups, and this remained true after adjusting for age. Similarly, there was no difference in operative time, pain scores, wound complications, or readmission rates between the groups. There was no difference in the primary outcome within the low or high BMI sub‐groups.

**Conclusion**: Use of Dermabond Prineo or traditional subcuticular suture provide similar patient satisfaction outcomes following cesarean delivery, with comparable rates of wound complications.

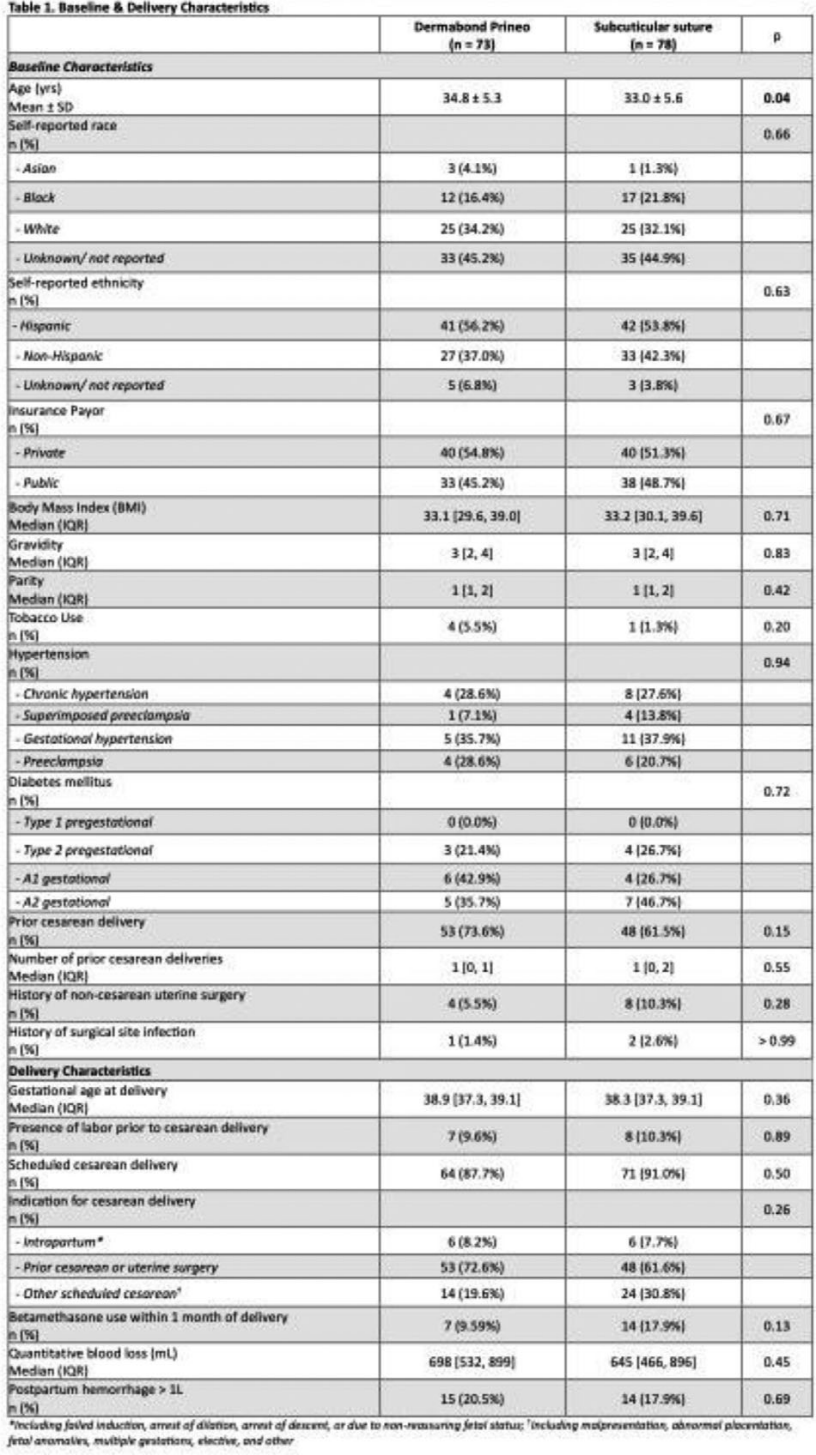

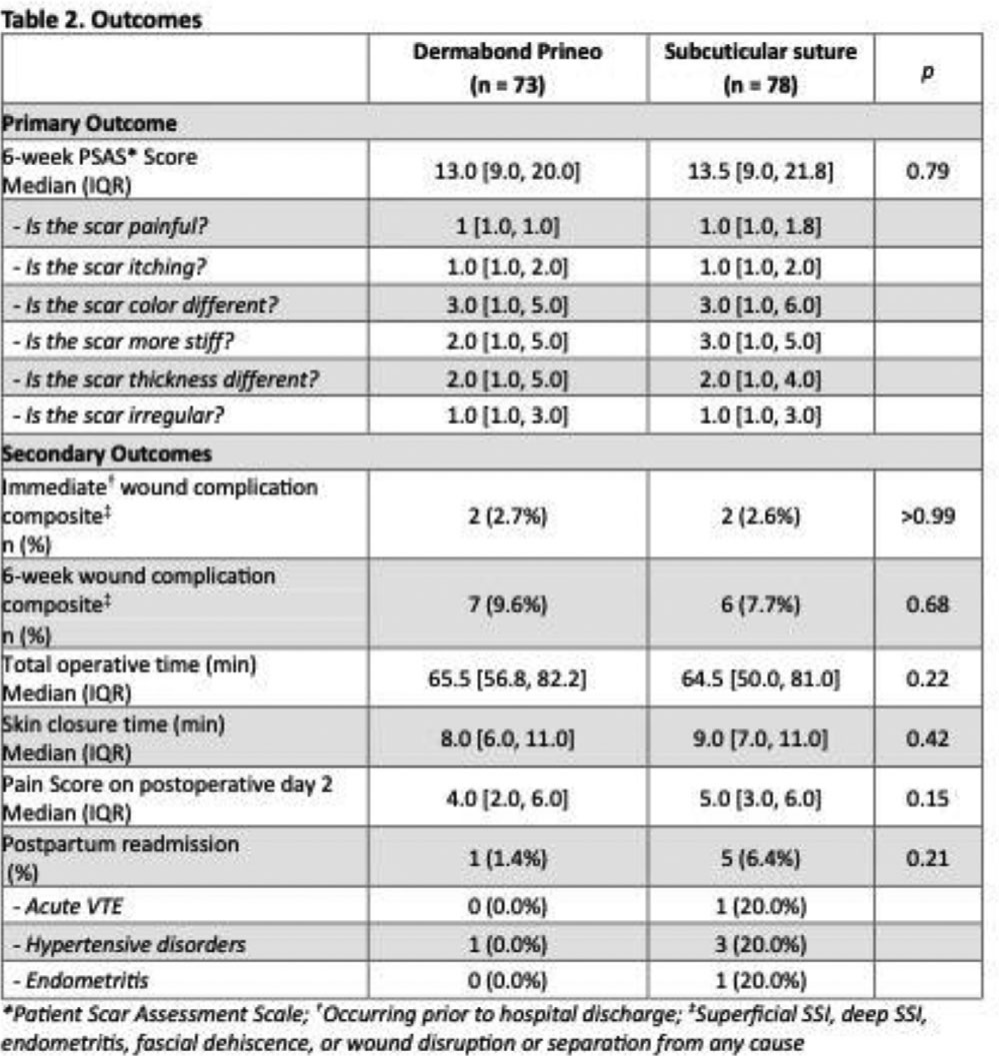

**9:15 AM – 9:30 AM**

## 42 | **DIP: An RCT of Diabetes Screening Immediately Postpartum After GDM**

Christine P. Field^1^; William A. Grobman^2^; Devra Mast^3^; Jiqiang Wu^3^; Shaylyn Vickers^3^; Veronica Csizmadia^3^; Anna Bartholomew^3^; Anna Chen^3^; Vidya Mullangi^3^; Steven Gabbe^3^; Elizabeth O. Buschur^3^; Erika F. Werner^4^; Mark B. Landon^1^; Kartik K. Venkatesh^1^

^1^The Ohio State University Wexner Medical Center, The Ohio State University Wexner Medical Center, OH; ^2^Warren Alpert Medical School of Brown University, Providence, RI; ^3^The Ohio State University Wexner Medical Center, Columbus, OH; ^4^Tufts University School of Medicine, Boston, MA

**Objective**: Patient adherence to postpartum glucose testing after gestational diabetes mellitus (GDM) remains suboptimal. We determined whether the frequency of testing differed when the oral glucose tolerance test (OGTT) was performed inpatient before delivery discharge (intervention) compared with outpatient within 12 weeks postpartum (standard care).

**Study Design**: Diabetes screening Immediately Postpartum (DIP) was a pragmatic, non‐blinded randomized controlled trial (RCT). Participants had a GDM diagnosis, received prenatal care and delivered at a tertiary care academic Midwestern medical center, and were enrolled postpartum during their delivery admission. The primary outcome was completion of a fasting 75‐g, 2‐hour OGTT by ≤12 weeks postpartum. Secondary outcomes included diagnosis of prediabetes or type 2 diabetes by OGTT and participant satisfaction per the adapted Diabetes Treatment Satisfaction Questionnaire.

**Results**: A total of 104 individuals with GDM were enrolled (52 intervention, 52 standard care). Demographics, unmet social needs, and clinical characteristics did not differ based on group assignment (Table 1). Postpartum visit completion was >90% in both groups. The frequency of postpartum diabetes screening was 92.3% in the inpatient group versus 26.9% in the outpatient group (relative risk: 3.43 [95% CI 2.18, 5.40] and risk difference: 65% [95% CI: 51%, 79%]) (Table 2). Prediabetes or type 2 diabetes was diagnosed among 50% in the inpatient group and 21.4% in the outpatient group (*p* = 0.05). Mean score on the Diabetes Treatment Satisfaction Questionnaire was higher (or better) in the inpatient compared with the outpatient group: 33.5 (SD 3.1) vs. 27.8 (SD 5.6) (*p* = 0.001).

**Conclusion**: Diabetes screening before delivery discharge resulted in over a three‐fold higher completion of postpartum glucose testing and improved patient satisfaction compared with current standard practice for GDM. This RCT supports recent guidelines that encourage the option of postpartum diabetes testing before delivery discharge.

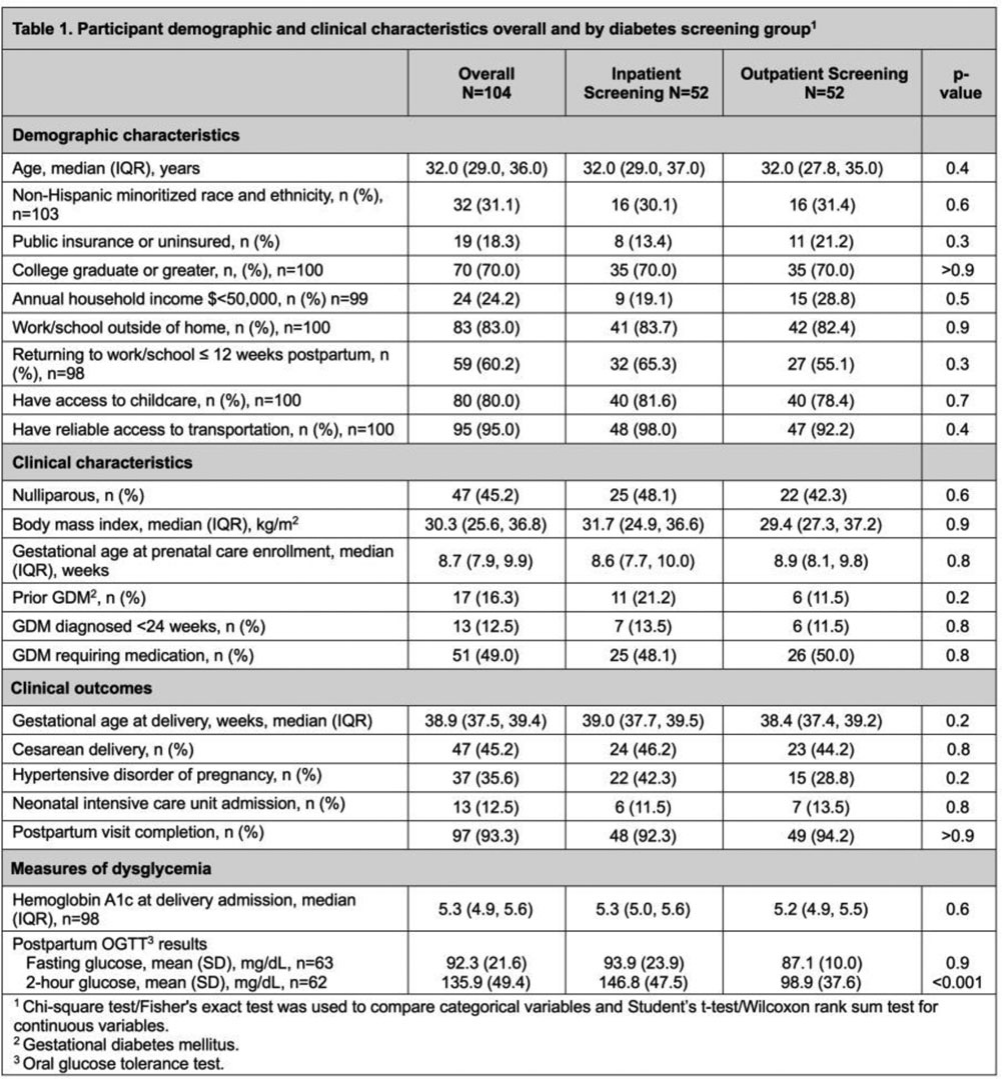

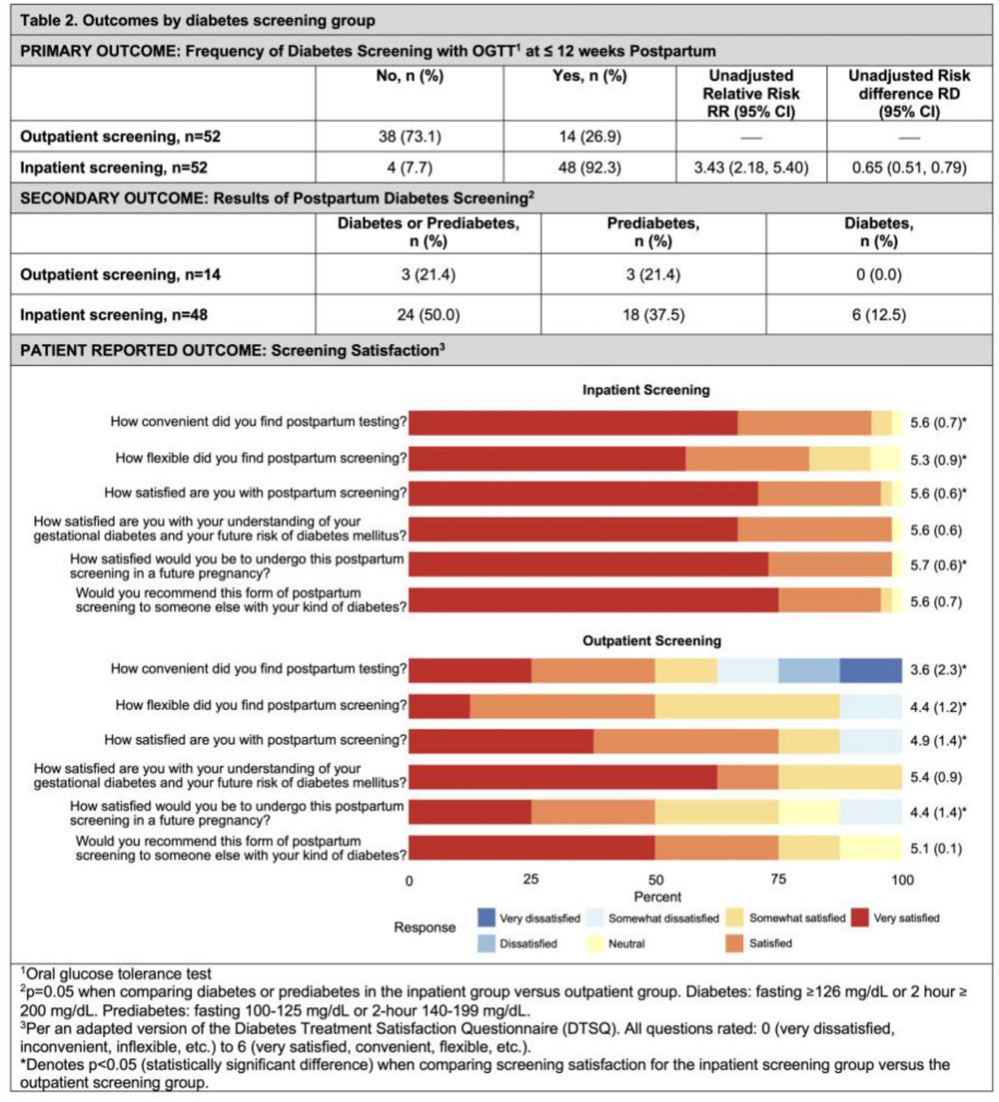

**9:30 AM – 9:45 AM**

## 43 | **TNF‐α Inhibition Improves Blood Pressure and Inflammation in a Preeclampsia Rat Model**

Baylee Brown^1^; Nathan Campbell^1^; Evangeline Deer^1^; Rachael Morris^1^; Ty Turner^1^; Alexandra Demesa^1^; Kennedy Cleveland^1^; Baoying Zheng^1^; Babbette LaMarca^2^

^1^University of Mississippi Medical Center, Jackson, MS; ^2^The University of Mississippi Medical Center, Jackson, MS

**Objective**: Preeclampsia (PE) is new‐onset hypertension with chronic inflammation and systemic endothelial dysfunction during pregnancy.  We've shown that CD4+ T helper cells contribute to PE phenotype through producing proinflammatory cytokines such as TNF‐α  . We have previously shown that etanercept, a soluble TNF‐α inhibitor, improves the PE phenotype in RUPP rats. Therefore, we hypothesize that etanercept will improve blood pressure, endothelial function, and inflammation in response to the adoptive transfer of placental PE CD4+ T cells into pregnant athymic nude rats.

**Study Design**: Placental CD4+ T cells were isolated from normal pregnant (NP) and PE patients at delivery. One million CD4+ T cells were injected I.P. into athymic nude rats on gestational day (GD)12. Etanercept (0.4 mg/kg) was given as an I.P. injection on GD15. Carotid catheters were implanted on GD18. Blood pressure (MAP), Preproendothelin, measured by RT‐PCR, and ET‐1 and TNF‐α were measured with ELISA on GD19. A one‐way ANOVA was used for statistical analysis.

**Results**: MAP increased with PE CD4+T cells to 120 ± 3 mmHg, *n* = 17, *p* < 0.05, compared to NP CD4+T cells (102 ± 4 mmHg, *n* = 12) which was blunted with Etanercept (110 ± 3 mmHg, *n* = 8). Renal ET‐1 was  lower in Etanercept treated rats (1.6 ± 034, *n* = 7) compared to PE recipients (3.4 ± 0.4 pg/mg protein). Placental ET‐1 was significantly increased with PE CD4+T cells (20.4 ± 0.7, *n* = 4) compared to NP CD4+Tcells (13.6 ± 2.4, *n* = 5) or Etanercept treated rats (10.8 ± 1.0 pg/mg protein, *n* = 5). Renal TNF‐α was significantly increased with PE CD4+ T cells (3.9 ± 0.3, *n* = 7) compared to NP recipients (3.1 ± 0.2 pg/mg protein, *n* = 6) and PE CD4+ T cells recipients treated with Etanercept (1.3 ± 0.2 pg/mg protein, *n* = 8).

**Conclusion**: TNF‐α blockade with Etanercept lowers blood pressure and multiorgan ET‐1, a marker of endothelial dysfunction and a hallmark of PE, caused by CD4+ T cells activation in preeclampsia.

**9:45 AM – 10:00 AM**

## 44 | **A Randomized Control Trial of Interventions to Increase Prenatal Tdap Acceptance Among Non‐Birthing Partners**

Laurie B. Griffin^1^; Natalie Passarelli^2^; Maxine Slater^3^; John Soehl^4^; Erica Hardy^3^; Melissa A. Clark^5^; Adam K. Lewkowitz^6^

^1^University of Utah, Cottonwood Heights, UT; ^2^Beth Israel Deaconess Medical Center, Boston, MA; ^3^Alpert Medical School of Brown University / Women & Infants Hospital of Rhode Island, Providence, RI; ^4^University of Wisconsin‐Madison, Madison, WI; ^5^Brown University School of Public Health, Providence, RI; ^6^Women & Infants Hospital of Rhode Island / Warren Alpert Medical School of Brown University, Providence, RI

**Objective**: Despite recommendations, many non‐birthing parents are not up to date on Tdap vaccination prior to delivery of their neonate. Among strategies to increase vaccination rates, education‐only initiatives have demonstrated inconsistent effects on vaccination rates. On‐site vaccination programs may be more efficacious by reducing access barriers not overcome by education alone. We sought to determine if prenatal in‐office vaccination opportunities increase non‐birthing partners vaccination rates prior to delivery.

**Study Design**: This randomized control trial enrolled non‐birthing partners who were not up to date on adult Tdap vaccination. Participants were recruited at a single outpatient prenatal clinic and randomized to (1) usual care, (2) vaccine education only, or (3) vaccine education plus in‐office vaccine administration during prenatal care.  We aimed to recruit 150 patients based on prior data suggesting a baseline Tdap vaccination rate 30% and expected 50% increase in the vaccine administration group. The primary outcome was non‐birthing partner Tdap vaccination rates postpartum. Secondary outcomes are in Table 1.  Risk ratios (RR) were calculated for the primary outcome.

**Results**: Of eligible individuals, 89% (150/168) participated and 93% (*n* = 140) completed the study. There were no sociodemographic differences between groups (Table 1). Participants in the in‐office administration group were 1.8 and 2.0 times more likely to receive Tdap vaccination during pregnancy compared to usual care  (RR 1.75, 95% confidence interval (CI) [1.04–2.94]) and education only (RR 2.04 95% CI [1.17–3.58]), while no difference existed between the usual care and education only groups (RR 0.86, 95% CI [0.44–1.65]). Overall,118 (84%) participants reported that prenatal in‐office vaccination programs would be desirable.

**Conclusion**: In‐office vaccination doubled prenatal Tdap vaccination rates, while education only was insufficient to increase vaccination rates compared to usual care. Studies focusing on implementation of this care delivery model are needed to determine sustainability and optimize vaccination opportunities.